# Perspectives of Neuro-COVID: Myasthenia

**DOI:** 10.3389/fneur.2021.635747

**Published:** 2021-02-26

**Authors:** Josef Finsterer, Fulvio A. Scorza

**Affiliations:** ^1^Klinik Landstrasse, Messerli Institute, Vienna, Austria; ^2^Disciplina de Neurociência, Escola Paulista de Medicine/Universidade Federal de São Paulo (EPM/UNIFESP), São Paulo, Brazil

**Keywords:** myasthenia, myastheic syndrome, COVID-19, SARS- CoV-2, pathophysiology

## Introduction

Managing immunologic disorders in the era of the severe acute respiratory syndrome coronavirus 2 (SARS-CoV-2) pandemic has become a challenge and is largely variable between specific disorders and between disciplines. This is also the case for neuroimmunologic disorders. Among the neuroimmunologic disorders, myasthenia gravis (MG) plays a specific role as it is clinically characterized by easy fatigability and transient or permanent muscle weakness. Fatigue and muscle weakness, however, are also key clinical features of the infection with SARS-CoV-2 [coronavirus disease 2019 (COVID-19)] why it can be challenging to differentiate between the two (1). Several questions arise regarding the management of MG patients who get infected with SARS-CoV-2. This opinion article aims at providing answers to several open questions as outlined below.

## Queries and Perspectives

### Does an Infection With SARS-CoV-2 Worsen or Exacerbate Myasthenia?

There are reports that described MG patients in whom the infection with SARS CoV-2 exacerbated MG (Table 1) (16), but there are also reports that show that MG remained stable throughout an infection with SARS-CoV-2 (Table 1) (3). According to a recent review about 16 MG patients experiencing a SARS-CoV-2 infection, MG deteriorated in eight patients and remained stable in the remaining patients (2). Among two of the eight patients experiencing deterioration of MG, a myasthenic crisis developed during the viral infection (2). Diagnosing a myasthenic crisis during COVID-19 can be challenging and requires strict adherence to established diagnostic criteria (17). A highly variable course and outcome has been also reported in a study of five MG patients experiencing COVID-19 (9). Whether the reaction of an MG patient to an infection with SARS-CoV-2 depends on the severity of the SARS-CoV-2 infection is unknown. Severity of COVID-19 is usually categorized as asymptomatic, mild, moderate, or severe. Severe COVID-19 may be further subdivided into COVID-19 not requiring mechanical ventilation and a second type requiring mechanical ventilation or extracorporeal membrane oxygenation. According to the few publications available, the reaction of MG to an infection with SARS-CoV-2 is independent of the severity of the viral infection. There are reports showing that mild or even subclinical COVID-19 may trigger exacerbation of MG (5), and there are reports showing that MG patients with severe COVID-19 do not experience an exacerbation of MG (5). The prevalence of exacerbations is also independent of the severity of the COVID-19 infection, as assessed by the CALL score (used for predicting the outcome of COVID-19 patients) (18).

**Table 1:** MG patients infected with SARS-CoV-2 reported as per the end of December 2020 [modified according to (2)].

**Age**	**Gender**	**MG type**	**MGTOA**	**DDC**	**Management**	**Outcome**	**References**
**MG diagnosed prior to COVID-19**							
42	F	AChR	CEI, PE, IG, S, IMT	Yes, crisis	PE stopped	Recovery	(3)
56	F	AChR	CEI, S, IG	Yes	IG, MV	Partial	(4)
38	F	AChR	CEI, S, IG, TE	Yes	S↑, IG↑	Recovery	(5)
65	M	AChR	CEI, S, IMT, PE	No	IG	Recovery	(5)
42	F	AChR	CEI, S, IG, TE	No	No change	Recovery	(5)
80	M	NR	IMT, IG	Yes, crisis	CEI, IG	Tracheotomy	(6)
66	F	MUSK	S, PE, IMT	No	NR	NR	(7)
36	F	NR	IMT, IG	Yes	IMT stopped	Recovery	(8)
36	F	NR	CEI, S, IG	No	NR	NR	(8)
55	M	NR	CEI	Yes	None	Recovery	(8)
25	M	NR	CEI, IMT	Yes	CEI ↑	Recovery	(8)
57	M	AChR	IMT	No	None	Recovery	(9)
64	M	AChR	IMT, S	No	None	Recovery	(9)
90	F	AChR	IMT, S, IG	No	None	Recovery	(9)
42	F	AChR	S	Yes	MV	MV	(9)
64	F	MUSK	S, IMT	No	None	Recovery	(9)
46	F	AChR	CEI, TE	Yes	CEI↑, CPT	Recovery	(10)
>60	NR	AChR	S	Yes	MV	Death	(11)
>60	NR	AChR	S	Yes	MV	Death	(11)
25	NR	AChR	S, IMT, IG	Yes	S, IMT, IG	Partial	(11)
45	NR	AChR	S, IMT	Yes	S↑, IMT	Partial	(11)
25	NR	NR	S, IMT	Yes	S	Partial	(11)
45	NR	NR	None	Yes	MV	Death	(11)
45	NR	AChR	S, IMT	Yes	MV, S, PE	Partial	(11)
45	NR	AChR	S, IMT	Yes	MV, S, PE	Partial	(11)
25	NR	AChR	S, IMT	Yes	MV, S, PE	Partial	(11)
25	NR	MUSK	S, RTX	Yes	MV, S, PE	Partial	(11)
25	NR	AChR	S, IMT	Yes	S, IMT	Recovery	(11)
>60	NR	NR	S	Yes	MV, S↑	Death	(11)
25	NR	NR	S, IMT	Yes	S↑	Partial	(11)
>60	NR	AChR	S, IG	Yes	MV, S↑	Partial	(11)
25	NR	AChR	S	Yes	MV, S↑	Recovery	(11)
70	M	AChR	CEI, S, IMT	Yes	MV, S↑, PE, IMT	Recovery	(12)
**Newly diagnosed MG triggered by SARS-CoV-2**							
64	M	AChR	None	NR	CEI, S	NR	(13)
68	M	AChR	None	NR	IG	NR	(13)
71	F	AChR	None	NR	PE	Favorable	(13)
21	F	AChR	None	NR	CEI, IG	Recovery	(14)
65	F	AChR	None	NR	CEI	Recovery	(15)

*CEI, choline esterase inhibitors; CPT, convalescent plasma therapy; DDC, deterioration of MG during COVID-19; IG, intravenous immunoglobulins; IMT, immune-modulating therapy; MGTOA, MG treatment on admission; MV, mechanical ventilation; PE, plasma exchange; RTX, rituximab; S, steroids. TE: thymectomy; NR, not reported*.

### How to Manage MG Therapeutically in Case of Concomitant SARS-CoV-2 Infection

#### MG Remains Stable

If the course of MG remains stable and unaffected by the viral infection, we should not adapt the therapeutic regimen. There is also no need to modify treatment in MG patients who are tested positive for SARS-CoV-2 but do not manifest clinically. In some patients, however, it may be useful to discontinue steroids or the immune-modulatory treatment to shorten viremia and accelerate viral clearance. This is because steroids are known to potentially prolong viremia and impair viral clearance in the acute stage (5). In the later stages of COVID-19, steroids may inhibit immune cell migration and chemokine production (5).

#### MG Exacerbates

If SARS-CoV-2 leads to exacerbation or worsening of MG, there is a need to adapt the therapeutic management. There are, however, no general recommendations guiding us with the adaptation of the therapy in case of exacerbation. Generally, exacerbation of MG is managed by increasing acetylcholine esterase inhibitors (ACEIs), by starting steroids or increasing the preexisting dosage, by starting or increasing the dosage of immune-modulating agents, by adding a biological (e.g., eculizumab), by adding intravenous immunoglobulins (IVIGs), or by applying plasma exchange (PLEX). As there are no general guidelines for the management of exacerbation of MG during an infection with SARS-CoV-2 available, we recommend to proceed in the same way as with an exacerbation of MG due to other causes. However, if the management of an exacerbation during COVID-19 is not successful, unconventional measures may be needed. Whether MG patients under immunomodulatory treatment are prone to acquire COVID-19 is unknown, but most neuromuscular societies recommend suspension of immune modulators if such a patient acquires COVID-19 (19). All such recommendations are unsupported by current data. However, modification of the immuno-suppression in transplanted patients with COVID-19 resulted in excellent recovery with intact graft function (8, 20). In a study of four MG patients experiencing COVID-19, the therapeutic regimen remained unchanged in two (8). In one patient, pyridostigmine was increased, and in the other patient, azathioprine was discontinued (Table 1) (8). Concerning the management of exacerbation of anti-MUSK–positive MG, for which rituximab is particularly effective, there are no consensus recommendations available. In a study conducted by Camelo-Filho et al., rituximab was discontinued in a single patient with MUSK-positive MG (Table 1).

#### Management of Myasthenic Crises Triggered by SARS-CoV-2

Treatment of myasthenic crises in SARS-CoV-2–infected MG patients has been outlined by Hoang et al. (20). The authors presented three cases with SARS-CoV-2–triggered myasthenic crises who profited from immune modulation with IVIGs or PLEX (20). Myasthenic crisis triggered by SARS-CoV-2 was also successfully treated with IVIGs in a 56-year-old woman who had been under pyridostigmine, prednisone, and IVIGs since 5 years prior (21). In case myasthenic crises triggered by SARS-CoV-2 leads to respiratory insufficiency, non-invasive, or invasive ventilation may be required. If spontaneous breathing is maintained, but oxygenation is insufficient, non-invasive positive-pressure ventilation via a continuous positive airway pressure (CPAP) mask is indicated. CPAP is a form of positive airway pressure (positive end-expiratory pressure) ventilation in which a constant level of pressure above atmospheric pressure is continuously applied to the upper airway. CPAP can be applied to the intubated or non-intubated patient. If spontaneous breathing is no longer guaranteed, mechanical ventilation is inevitable.

### Is There a Need to Avoid Antivirals, Neutralizing Antibodies, Convalescent Plasma, Immune Modulators, Cytokine Inhibitors, or Antibiotics Frequently Applied to COVID-19 Patients?

As with any other compound administered to MG patients, all drugs given for the treatment of SARS-CoV-2 need to be evaluated for their potential to exacerbate or deteriorate MG. Drugs from which it is known that they potentially deteriorate MG and which are frequently given to COVID-19 patients include steroids, chloroquine (22, 23), and azithromycin (may even trigger myasthenic crises) (23). Interleukin 6 (IL-6) inhibitors, such as tocilizumab, sarilumab, or siltuximab, do not seem to deteriorate myasthenia. There are even indications that IL-6 inhibitors exhibit a beneficial effect on MG (9). However, no systematic studies have been carried on this issue so far. Remdesivir, lopinavir, and ritonavir have not been reported to exacerbate MG.

### Does the Management of SARS-CoV-2–Infected MG Patients Depend on the Underlying Immune Mechanism?

Autoimmune MG can be associated with different types of autoantibodies. Most frequently, these antibodies are directed against the acetylcholine receptor (AchR). More rarely, antibodies directed against MUSK, LRP4, agrin, or titin are responsible for MG. The question raised cannot be reliably answered as there are not sufficient data available, but we do not see the need to deviate from the recommended regimens for treating these different subtypes of MG in case of an exacerbation during a SARS-CoV-2 infection as long as MG does not deteriorate. So far, only few patients with MG due to anti-MUSK antibodies and COVID-19 have been reported (7, 9).

### Can SARS-CoV-2 Trigger New MG?

This question can be definitively answered with “yes” as there is increasing evidence that COVID-19 not only can exacerbate MG, but also can trigger the development of new MG. In a recent study, Restivo et al. presented three patients who newly developed MG 5–7 days after onset of COVID-19 (13). Clinical manifestations of MG were mild in all three patients (13). In all three patients, MG was due to AchR antibodies (13). All three patients profited from pyridostigmine, steroids, IVIGs, or PLEX. The outcome was favorable in all of them. Restivo et al. suspected that epitope homology between surface proteins of the virus and the AchR could be responsible for the development of MG (13). New postinfectious MG has been also reported in a 21-year-old woman with mild COVID-19 who developed ptosis and double vision already during the 10 days of COVID-19 (14). She was tested positive for AchR antibodies, and MG manifestations resolved under pyridostigmine and IVIG. A fifth case with new, postinfectious ocular myasthenia has been reported by Srivastava et al. (15). The patient was a 65-year-old woman who developed ptosis and double vision 2 weeks after onset of mild COVID-19 (15).

### Are MG Patients Receiving Immune-Suppressive Therapy Protected Against Complications of COVID-19?

Steroids are known to prolong viremia and to impair viral clearance in the acute stage (5). In the later stages of the infection, however, they may inhibit immune cell migration and chemokine production (5). Thus, it can be speculated that steroids worsen the infection in the early stage but mitigate the immune response in the later stages. The effect of azathioprine, mycophenolate mofetil, cyclosporine, methotrexate, tacrolimus, cyclophosphamide, and rituximab on the virus infection is unknown. There is one case report about an immunosuppressed MG patient who did get infected from her infected relatives who developed mild COVID-19 (25). The reason why the patient did not get infected despite close contact with her husband and son during 6 days remains elusive, but it can be speculated steroids and mycophenolate mofetil, she was regularly taking, had a protective effect. Whether MG patients having undergone previous thymectomy have a milder course of COVID-19 and a better outcome compared to MG patients without thymectomy remains elusive, as not enough data about this issue have been published yet. A 86-year-old woman with MG having undergone thymectomy 1.5 years prior to COVID-19 recovered completely after mechanical ventilation, PLEX, and stress-dosage intravenous steroids (16).

### Should Non-infected MG Patients Receive a Prophylaxis With Chloroquine?

As chloroquine is ineffective to protect against COVID-19 and as chloroquine is myotoxic, there is no need to apply this compound to SARS-CoV-2–negative MG patients as a prophylaxis. Because the inherent potential of causing myopathy, we generally suggest not to apply chloroquine to MG patients. There is even one report showing that chloroquine may trigger the development of a myasthenic syndrome (26). Chloroquine may even exacerbate MG (22). There is also no need to apply azithromycin as the drug is not effective for COVID-19 and carries the potential to be myotoxic as well (27). This is also the case with linezolid and meropenem (27). From remdesivir, ritonavir, and lopinavir, it is known that they occasionally trigger rhabdomyolysis (28).

## Discussion

Management of MG patients who get infected with SARS-CoV-2 depends on the reaction of MG to the infection. If MG deteriorates, anti-MG therapy has to be modified. If MG remains stable, there is no need for a change. In the early stages of COVID-19, however, steroids should be discontinued. It is important to avoid any MG deteriorating medication in MG patients experiencing COVID-19. There is increasing evidence that SARS-CoV-2 may trigger the development of new MG. possibly through molecular mimicry between surface virus epitopes and the AchR. However, the virus may initiate autoimmunity not only by molecular mimicry, but also by epitope spreading, bystander activation, or immortalization of infected B cells (29). MG patients should not receive any prophylaxis for a putative SARS-CoV-2 infection as there is generally no prophylaxis against the virus infection currently available. Whether MG patients under immuno-suppression are safer from being infected compared to those who do not receive immuno-suppression remains speculative, but it is conceivable that the cytokinetic storm after the acute viremia may be less strong than in subjects not receiving such a treatment. As COVID-19 patients may develop myopathy, there is a need to delineate MG from myopathy due to COVID-19, from myopathy due to drugs given for the treatment of COVID-19, and from critically ill myopathy. This can be achieved by demonstration of the beneficial effect of ACEIs, by measuring AchR or MUSK antibody titers, by repetitive nerve stimulation, and by single-fiber electromyography. Whether MG patients develop a more severe course of COVID-19 than non-MG patients, as has been recently forwarded (11), remains uncertain. In this study of 15 MG patients from São Paolo experiencing COVID-19, 87% required transfer to the intensive care unit, 73% required artificial ventilation, and 30% of these patients died (11). Thus, the notion that there is no evidence of an elevated risk of morbidity and aggravation of COVID-19 in MG patients, irrespective of the treatment they receive, as recently stated (30), cannot be confirmed. In a recent retrospective study of 91 patients with COVID-19, 40% experienced exacerbation of MG requiring rescue therapy with IVIGs, PLEX, or stress steroids (31).

In conclusion, MG deteriorates in half of the patients experiencing COVID-19. In a quarter of these patients, deterioration manifests as myasthenic crisis. Anti-MG therapy should be modified only if MG deteriorates during the infection. SARS-CoV-2 not only can exacerbate MG, but also can trigger new, postinfectious MG. Drugs given for COVID-19 but potentially harmful for MG patients include steroids, chloroquine, and azithromycin. Drugs deteriorating MG should be absolutely avoided in MG patients experiencing COVID-19.

## Author Contributions

All authors listed have made a substantial, direct and intellectual contribution to the work, and approved it for publication.

## Conflict of Interest

The authors declare that the research was conducted in the absence of any commercial or financial relationships that could be construed as a potential conflict of interest.
